# 2-Phosphonobutane-1,2,4,-Tricarboxylic Acid (PBTC): pH-Dependent Behavior Studied by Means of Multinuclear NMR Spectroscopy

**DOI:** 10.3390/molecules27134067

**Published:** 2022-06-24

**Authors:** Jerome Kretzschmar, Anne Wollenberg, Satoru Tsushima, Katja Schmeide, Margret Acker

**Affiliations:** 1 Helmholtz-Zentrum Dresden-Rossendorf, Institute of Resource Ecology, 01328 Dresden, Germany; s.tsushima@hzdr.de (S.T.); k.schmeide@hzdr.de (K.S.); 2 Radiochemistry and Radioecology, Technical University Dresden, 01062 Dresden, Germany; anne.wollenberg@tu-dresden.de; 3 Central Radionuclide Laboratory and Radiation Protection, Technical University Dresden, 01062 Dresden, Germany; margret.acker@tu-dresden.de; 4 International Research Frontiers Initiative, Institute of Innovative Research, Tokyo Institute of Technology, Meguro, Tokyo 152-8550, Japan

**Keywords:** PBTC, NMR, DFT, protolysis, speciation, thermodynamic constant

## Abstract

Although 2-phosphonobutane-1,2,4,-tricarboxylic acid, PBTC, has manifold industrial applications, relevant and reliable data on the protonation of PBTC are poor. However, these data are critical parameters for ascertaining PBTC speciation, especially with regard to a sound structural and thermodynamic characterization of its metal ion complexes. A rigorous evaluation of pH-dependent ^1^H, ^13^C, and ^31^P chemical shifts along with accessible scalar spin–spin coupling constants (*J*) was performed in order to determine the p*K*_a_ values of PBTC in 0.5 molal NaCl aqueous solution by means of nuclear magnetic resonance (NMR) spectroscopy. The phosphonate group revealed p*K*_a_ values of 0.90 ± 0.02 and 9.79 ± 0.02, and the p*K*_a_ values associated with the carboxylic groups are 3.92 ± 0.02, 4.76 ± 0.03, and 6.13 ± 0.03. Supported by DFT-calculated structures revealing strong intramolecular hydrogen bonding, the sequence of deprotonation could be unambiguously determined.

## 1. Introduction

**Figure 1:**
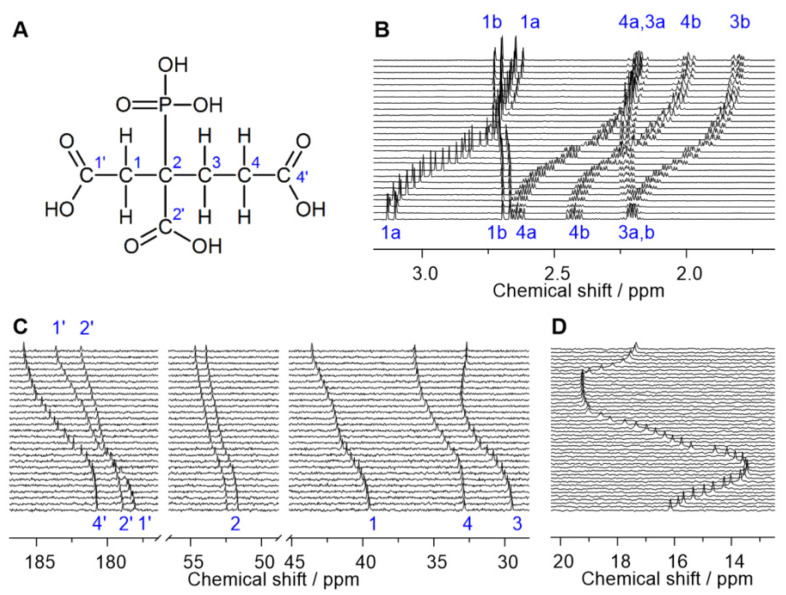
(**A**) Structure of PBTC together with atomic labeling used throughout this work. (**B**–**D**) NMR pH-titration series of PBTC in 0.5 molal NaCl solutions. (**B**) ^1^H{^31^P} NMR and (**C**) ^13^C{^1^H} NMR spectra of 25 mM PBTC in the pH range 2–8, and (**D**) ^31^P{^1^H} NMR spectra of 1 mM PBTC in the pH range 0–11; pH values increase from bottom to top with increments of 0.2 to 0.4 pH units. For clarity, only spectral regions of interest are shown.

Organophosphonates are multipurpose basic chemical compounds. Such products are used in cooling water systems, desalination plants, in the paper, textile, and cement industries as well as in detergents [1]. They act primarily as complexing agents to prevent salt precipitation (antiscalants) and as bleach stabilizers. One of the most widely used organophosphonates is 2-phosphonobutane-1,2,4-tricarboxylic acid, PBTC (Figure 1A) [2,3,4,5]. The combination of phosphonic acid and carboxyl groups makes PBTC an effective dispersant and highly powerful complexing agent for various metal ions (including Ca^2+^, Mg^2+^, Zn^2+^, Al^3+^, Fe^3+^) and radionuclides [6,7,8,9]. Due to these properties, PBTC is used in a wide range of technical applications and has been extensively studied in numerous publications and patents. For example, PBTC serves as an efficient long-term retarder in concrete, as a corrosion inhibitor in reinforced concrete and steel, and as a scale inhibitor in water treatment and circulating cooling water systems [10,11].

Regardless of the numerous uses of PBTC in industrial applications, relevant and reliable thermodynamic data on the protonation of PBTC are scarcely available. Protonation constants are necessary thermodynamic parameters for ascertaining PBTC speciation, especially with regard to a thorough structural and thermodynamic characterization of its metal ion complexes. In particular, the protonation constants p*K*_a1_ and p*K*_a5_ show a lack of consistency among the published values (Table 1) that cannot be explained by factors such as different media and ionic strengths. The first p*K*_a_ values were published in 1982 in a data sheet for PBTC solution by Bayer AG known under the trade name BAYHIBIT^®^AM (data available in [12]). Additionally, protonation constants estimated by calculation were released by the European Chemicals Agency (ECHA) [13]. Salvado et al. [8] determined the p*K*_a_ values by potentiometric pH titrations in 0.5 M NaNO_3_ at 25 °C; however, this study used commercially available PBTC without any further characterization or purifications. Kornev et al. [14] also lacks information regarding the origin and purity of the PBTC solution used for potentiometric titrations.

NMR spectroscopic techniques have been proven useful for p*K*_a_ determination for a large variety of molecules [15,16,17,18]. The advantage of these techniques is that they provide structure-related information and cover a wider pH range. For example, determination of a p*K*_a_ value of about 1 is hardly feasible in potentiometric or calorimetric titration as detection of the abstracted proton is poor against background H^+^ concentrations ranging between 0.1 and 1 M, corresponding to pH values between 1 and 0, respectively. Especially for phosphorous-containing compounds, the value of ^31^P NMR-based pH-titrations has been clearly demonstrated by the work of the Hägele group [19,20,21,22].

**Table 1:** Overview and comparison of PBTC p*K*_a_ values along with method and conditions.

p*K*_a_					Method	Conditions ^1^	Reference
1	2	3	4	5			
**PBTC**							
1.08 ± 0.42	3.99 ± 0.21	4.44 ± 0.10	4.99 ± 0.14	8.59 ± 0.50	estimation by calculation	water, 20 °C	[13]
1.8	4.0	4.9	6.8	10.8	not specified	not specified	[12] ^2^
2.20 ± 0.03	4.03 ± 0.01	4.97 ± 0.01	6.68 ± 0.01	10.19 ± 0.01	potentiometric titration	0.1 M NaCl, (20 ± 2) °C	[14]
3.865	4.145	5.17	6.83	9.038	potentiometric titration	0.5 M NaNO_3_, 25 °C	[8]
0.90 ± 0.02	3.92 ± 0.02	4.76 ± 0.03	6.13 ± 0.03	9.79 ± 0.02	NMR-based titration	0.5 m NaCl, (25 ± 1) °C	this work
**(*R,S*)/(*S,R*) PPTC ^3^**							
1.12 ± 0.02	3.75 ± 0.01	4.78 ± 0.01	6.63 ± 0.01	9.13 ± 0.01	NMR-based titration	not specified	[20]
**(*R,R*)/(*S,S*) PPTC ^3^**							
1.02 ± 0.07	3.37 ± 0.03	4.87 ± 0.03	6.30 ± 0.02	9.19 ± 0.02	NMR-based titration	not specified	[20]

^1^ M and m denote molar and molal concentrations, respectively. ^2^ Product data sheet of BAYHIBIT^®^AM 1982, values published in [12]. ^3^ 1-Phosphonopropane-1,2,3-tricarboxylic acid, occurring as diastereomeric pairs of enantiomers.

Within the framework of studies on the release of the long-term retarder PBTC from aged cement phases and related investigations on the complexation behavior towards metal ions under (hyper-)alkaline and highly ionic conditions, we determined p*K*_a_ values of PBTC. Therefore, we performed solution NMR spectroscopic pH-titration to determine PBTC’s pH-dependent speciation and concomitant structural changes (conformation). Structural aspects such as hydrogen bonds and ambiguities in the deprotonation sequence were clarified by DFT-optimized structures.

## 2. Results

### 2.1. NMR Signal Assignment

Unambiguous NMR signal assignment was achieved by heteronuclear single-quantum coherence (HSQC) and heteronuclear multiple-bond correlation (HMBC) between ^1^H and ^13^C, as well as ^1^H and ^31^P nuclei, allowing for determination of the H, C, and P atomic connectivity. Exemplary spectra are provided in the Supplementary Materials, Figure S1.

In principle, the three methylene groups are diastereotopic, resulting in distinct ^1^H resonances of either proton in one CH_2_ group as clearly seen in the H,C-HSQC spectra. The ^31^P nucleus gives rise to characteristic heteronuclear scalar spin–spin couplings via *n* bonds (*^n^J*), observable for certain signals in both ^1^H and ^13^C spectra. In this work, only the absolute values are quoted, as we do not consider the sign of *J* in our analysis. Examination of the ^1^H spectra reveals typical *vicinal* (^3^*J*_H,H_) coupling between the adjacent methylene groups, H3(a/b) and H4(a/b). In addition, the protons attached to the same carbon are non-equivalent, as they show *geminal* (^2^*J*_H,H_) coupling. As is typical for ^31^P-related scalar couplings, the commonly observed order is ^1^*J* >> ^3^*J* > ^2^*J* > ^4^*J*, frequently rendering the latter two often unresolved. The carboxylic carbons can be assigned based on their appearance. That is, C4′ is a singlet as the ^4^*J*_C,P_ is too small to be observed. Accordingly, the carboxyl ^13^C doublet with the larger splitting is due to C1′ (^3^*J*), whereas that with the smaller splitting arises from C2′ (^2^*J*). Correspondingly, among the methylene carbons, the signal associated with C4 is the only one that consistently appears as a doublet (^3^*J*), whereas those of C1 and C3 do so only occasionally (^2^*J*). The ^2+3^*J*_C,H_ resolved in the HMBC spectrum confirm this assignment.

Scalar coupling also occurs between ^31^P and ^1^H nuclei, as seen in the H,P-HMBC spectrum and the projected ^1^H and ^1^H{^31^P} spectra (Supplementary Materials, Figure S1A). Again ^3^*J* > ^4^*J* holds true, allowing for discrimination between H3(a/b) (^3^*J*_H,P_) and H4(a/b) (^4^*J*_H,P_), where ^1^H signals of the latter reveal no significant alterations upon acquisition with ^31^P-decoupling. Since H1(a/b) show no coupling to the other methylene protons, their assignment is straightforward.

In particular, the signal splitting of C2 (^1^*J*_C,P_~120 Hz), C2′ (^2^*J*_C,P_~3.5 Hz), C1′ (^3^*J*_C,P_~12 Hz), and C4 (^3^*J*_C,P_~8 Hz) is a recurring spectral feature. The latter three coupling constants are revealed to be pH-dependent and thus reflect the modulations in conformation upon changing speciation along the pH-titration series (vide infra). Of these, the two ^3^*J*_C,P_ show Karplus-like curves, indicating extensive charge-induced alterations in (P–C–C–C) dihedral angles.

### 2.2. pK_a_ Determination from NMR pH-Titration Series

**Figure 2:**
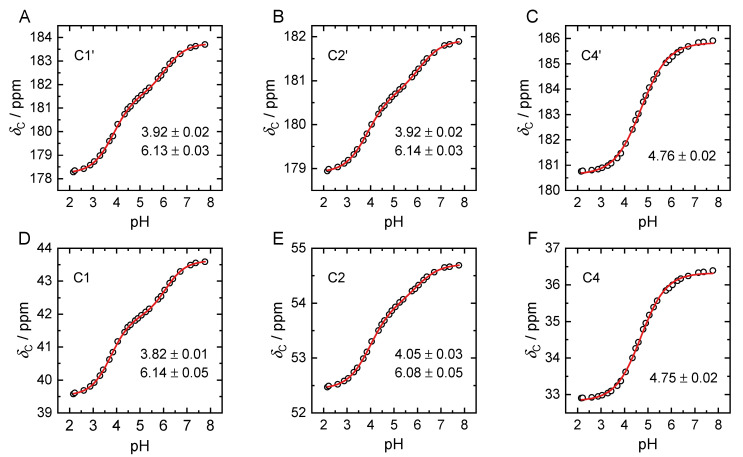
Graphs of pH-dependent ^13^C NMR chemical shift values (black circles) obtained from pH-titration series of 25 mM PBTC in 0.5 molal NaCl solutions. Graphs respectively correspond to carboxyl carbons C1′ (**A**), C2′ (**B**), C4′ (**C**), and methylene carbons C1 (**D**), C2 (**E**), C4 (**F**). The p*K*_a_ values obtained from sigmoidal (bi-)dose–response fits (red lines) are stated with each subfigure.

^1^H, ^13^C, and ^31^P NMR spectra of pH-titration series in 0.5 molal NaCl H_2_O solution are depicted in Figure 1B–D, respectively. An analogous set of spectra for a pD-titration series of 0.35 M PBTC in D_2_O solution without background electrolyte is given in the Supplementary Materials, Figure S2. The graphical evaluation of the pH-dependent ^13^C NMR chemical shift values is given in Figure 2.

**Figure 3:**
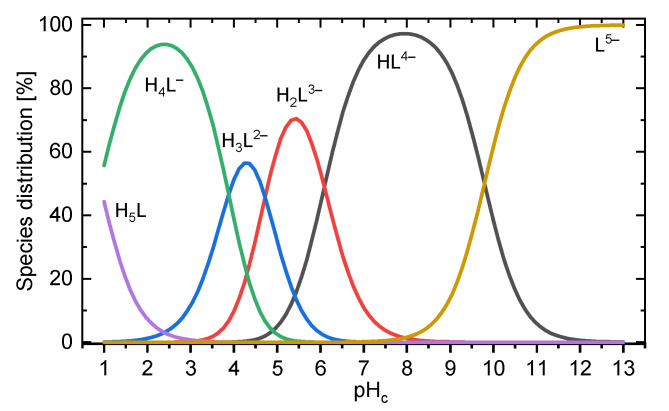
Species distribution diagram calculated for 1 mM of total PBTC based on the p*K*_a_ values obtained in this work (*I_m_* = 0.5 m NaCl, *T* = 25 °C).

Upon increasing pH, the ^13^C signals generally shift downfield [23]. The ^31^P signal shows a more complex behavior. Similar to other phosphonate compounds, proton abstraction at the phosphonic group itself is associated with upfield shifts and occurs in the strongly acidic pH range as well as in the alkaline pH regime. The intermediately observable downfield shift between pH 2 and 7 is associated with the consecutive deprotonation of the carboxylic functional groups. Between pH 7 and 8 the signal position remains constant, mirroring PBTC’s unchanged speciation in that range (Figure 3). The signals of the ^1^H nuclei show peculiar behavior as they are sensitive to both changing speciation and alterations in the molecule’s conformation accompanied with variations in chemical (spatial) environments (vide infra).

Evaluation of the ^31^P NMR pH-titration data in order to determine associated p*K*_a_ values is straightforward and, therefore, *δ*_P_ vs. pH plots along with corresponding sigmoidal dose–response fits are shown in the Supplementary Material, Figure S3. Thus, the determined protolysis constants are p*K*_a1_ = 0.90 ± 0.02 and p*K*_a5_ = 9.79 ± 0.02.

Concerning the PO_3_H_2_ group, the p*K*_a_ values associated with the COOH groups could also be determined with small statistical uncertainty, with corresponding fitting parameters provided in Table S1 of the Supplementary Material. However, for two out of the three obtained p*K*_a_ values, assignment to the corresponding COOH group was not straightforward. The p*K*_a3_ value of 4.76 ± 0.03 can be unambiguously attributed to the C4′ carboxylic group (Figure 2C,F). In the case of p*K*_a2_ and p*K*_a4_, determined as 3.92 ± 0.02 and 6.13 ± 0.03, respectively, assignment to the two remaining carboxylic groups C1′ and C2′ is difficult, see Figure 2A,D vs. B,E. Upon application of sigmoidal bi-dose–response fit functions, the pH-dependent chemical shift data of both these carboxylic groups as well as of their adjacent carbons (C1 and C2, respectively) reveal two inflections at virtually identical pH values. Obviously, the individual pH-dependent chemical shift values are strongly correlated. The individual line widths (signal width at half amplitude) are insignificant. As a further NMR spectroscopic quantity, the ^13^C–^31^P scalar couplings were considered. Among these *^n^J*_C,P_ coupling constants, the ^3^*J*_C1′,P_ (vide infra) can be fitted by a sigmoidal bi-dose–response fit function (Supplementary Materials, Figure S4), and reveals almost the same two inflection points already determined from the chemical shift data, viz. 4.01 and 6.16.

Table 1 (vide supra) gives a concise summary of the few PBTC titration literature data. Figure 3 shows a species distribution according to the p*K*_a_ values determined in this work, obtained in 0.5 m NaCl aqueous solution.

### 2.3. DFT-Calculated Deprotonation Sequence

Relative energies of DFT-optimized molecular structures of differently protonated H*_n_*L*^n^*^−5^ (*n* = 0, 1, 2, 3, 4, 5) species are summarized in Table 2, and provide further insight into the actual order of deprotonation in this system.

**Figure 4:**
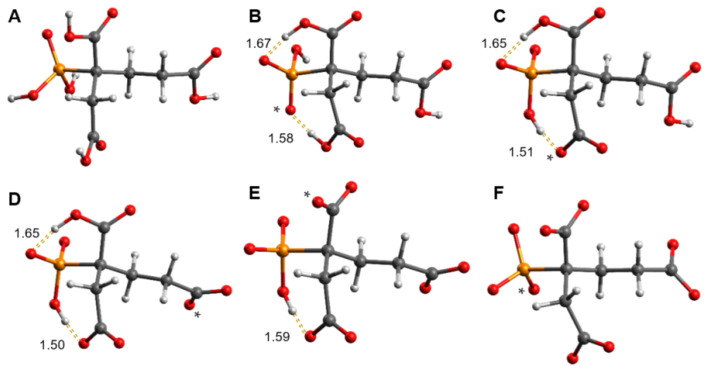
DFT-optimized structures of H*_n_*PBTC*^n^*^−5^ species of lowest energy (*n* = 5 through 0, corresponding to consecutive deprotonation, (**A**–**F**), respectively), according to Table 2. Sites of respective deprotonation are indicated by *. Dashed lines indicate intramolecular hydrogen bonds, with OH⋯O distances given in Å.

DFT-calculated energies clearly demonstrate that the phosphonic acid group is associated with PBTC’s first and last deprotonation step. Deprotonation of H_4_L^−^ at carboxylic groups 2′ and 4′ results in structures with higher energy than for proton abstraction at 1′. Thus, the second deprotonation, associated with p*K*_a2_, is favored for carboxylic group 1′. For the trianionic species, the structure with the proton abstracted from carboxylic group 4′ is somewhat lower in energy, hence associated with p*K*_a3_. Therefore, p*K*_a4_ refers to carboxylic group 2′. DFT-calculated structures of the respective deprotonated species of lowest energy are compiled in Figure 4.

### 2.4. pH-Dependent Scalar Spin–Spin Coupling Constants

**Figure 5:**
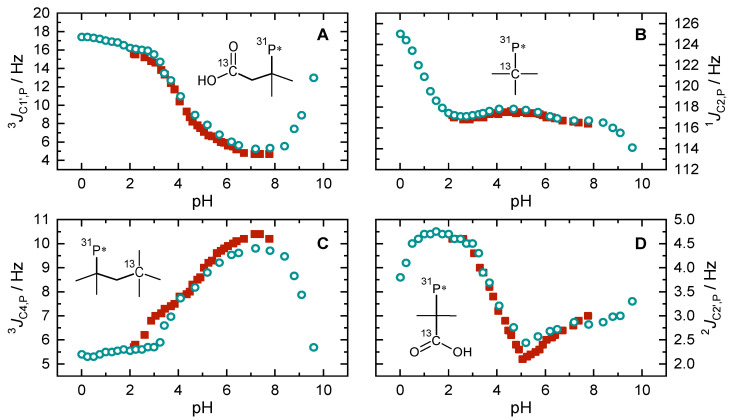
Graphs showing scalar ^13^C–^31^P spin–spin coupling constants (absolute values) in dependence on pH/pD. (**A**) ^3^*J* (C1′–P), (**B**) ^1^*J* (C2–P), (**C**) ^3^*J* (C4–P), and (**D**) ^2^*J* (C2′–P). Red squares correspond to pH-titrations series of 25 mM PBTC in 0.5 molal NaCl 90/10 (*v*/*v*) H_2_O/D_2_O solutions, and blue circles correspond to pD-titration series of 350 mM PBTC in D_2_O solution without background electrolyte. Inserts sketch the respective considered coupling pathways. P* denotes the phosphonate group.

Scalar spin–spin couplings were determined from signal splitting of C1′, C2, C4, and C2′ (Figure 5A–D) for two titration series, i.e., 25 mM PBTC and 0.5 molal NaCl in 90/10 (*v*/*v*) H_2_O/D_2_O (red squares) as well as 350 mM PBTC in pure D_2_O solutions (blue circles). We are aware that H and D possess non-negligible isotope effects. However, since superposition of the data from both series indicates consistent behavior, and since the D_2_O series covers a wider data range than the H_2_O series, the following considerations focus primarily on the D_2_O series.

Initially, at pD = 0, PBTC is fully protonated and hence constitutes a neutral molecule that notably retains good solubility, even for concentrations as high as 350 mM. ^1^*J*_C2,P_ shows a steady decrease over the course of either of the two PO_3_H_2_ deprotonation steps, that is in contrast to almost no variations while titrating the COOH groups (Figure 5B). The curves of the two ^3^*J*_C,P_ involving C1′ and C4, Figure 5A,C, respectively, illustrate almost opposite progression during pD titration. In strongly acidic media, ^3^*J*_C1′,P_ displays its maximum of ~18 Hz while ^3^*J*_C4,P_ reveals its minimum of ~5.5 Hz. Up to pD~3, minimal changes are observed. Upon further increase of the pD to about 7, ^3^*J*_C1′,P_ passes its minimum of ~5 Hz, whereas ^3^*J*_C4,P_ is at its maximum of 10.5 Hz. Finally, in increasingly alkaline media and with the abstraction of the second phosphonate proton, the trends of both curves return to similar values as in acidic solution. The ^2^*J*_C2′,P_ is, in principle, small and so are its pD-dependent variations. Nevertheless, the curve discloses a clear minimum (~2 Hz) at pD = 5.

**Figure 6:**
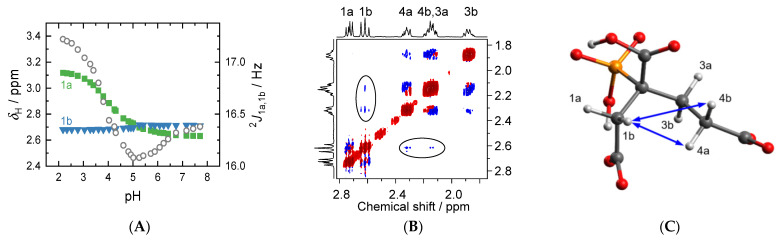
(**A**) ^1^H chemical shift (left *y*-axis) of H1a and H1b (green squares and blue triangles, respectively) as well as their *geminal* coupling constant (right *y*-axis, absolute values, open circles) in dependence on pH, as determined from ^1^H{^31^P} NMR spectra shown in Figure 1B. (**B**) NOESY spectrum (500 ms mixing time) of PBTC pH 4.4 solution, and (**C**) DFT-calculated structure of H_3_L^2−^, indicating the non-trivial NOE contacts by ellipses and arrows, respectively.

Among the ^1^H-related coupling constants, the ^2^*J*_1a,1b_ (Figure 6A) is easiest to evaluate and of particular interest. Its absolute value is relatively large, even for a *geminal* coupling constant. This is mainly due to the proximity of the H-atoms to the carbonyl carbon of the carboxylic group C1′, as previously observed for glycine residue (~18 Hz) or citrate (~16 Hz) [24,25], and reinforced by the strong electron withdrawing effects from the close phosphonate and carboxyl groups. Analogous to the ^2^*J*_C2′,P_ discussed above, the pH-dependent variance is small but smooth and significant, showing a minimum at pH = 5 as well. The pH-dependent behavior of the signals associated with H1(a/b) is very different (see Figure 1B and Figure 6A). 1a shows a drastic change in chemical shift between pH 3 and 5, whereas the signal due to 1b is nearly independent of pH.

## 3. Discussion

As seen from Table 1, the available protolysis data are heterogeneous and ambiguous. Our obtained p*K*_a_ values appear reasonable overall and are comparable to other phosphonic acids. For instance, in phosphonoacetic acid and 2,4-diphosphonobutane-1,2-dicarboxylic acid (DPBDC), p*K*_a1_ values associated with H^+^ abstraction from the phosphonic acid group are 0.78 and 1.07, respectively [19]. The two carboxyl groups in DPBDC reveal corresponding p*K*_a3_ and p*K*_a4_ values of 4.82 and 7.05 [19], which are also in accordance with our determined figures. Considering the closely structurally related 1-phosphonopropane-1,2,3-tricarboxylic acid (PPTC, occurring as diastereomeric pairs of enantiomers, included in Table 1), both the phosphonic acid and carboxyl groups show remarkably similar p*K*_a_ values [22], further corroborating our figures.

The prediction of the deprotonation sequence for the carboxyl groups is not easily accomplished through purely chemical reasoning. Based on the structure of the PBTC molecule, on the one hand, one might expect that 4′ COOH possesses the largest p*K*_a_ value since it is distant from the other electron-withdrawing functionalities. On the other hand, for the partially ionized species, intramolecular hydrogen bonds play an important role, while for the completely protonated acid the DFT-calculated structure revealed no intramolecular hydrogen bonds. The latter finding is in agreement with the crystal structure of PBTC acid where hydrogen bonds are intermolecular [26].

For DPBDC, Hägele proposed intramolecular hydrogen bridges in protolytic species H_3_L^3−^ to HL^5−^ with formation of a seven-membered ring between the phosphonate and the carboxyl groups in γ-position [19]. Our DFT-calculated structures (Figure 4) corroborate intramolecular hydrogen bonds with formation of six- and seven-membered rings involving PBTC’s phosphonic acid group and the carboxylic groups 1′ and 2′ in the γ- and β-positions, respectively. The latter of these appears to persist for the PBTC species H_4_L^−^ through H_2_L^3−^ (Figure 4B,D). For species H_4_L^−^, 1′ COOH is a hydrogen bond donor with one of the phosphonate oxygens as acceptor, whereas for species H_3_L^2−^ through HL^4−^ (Figure 4C,E) 1′ COO^−^ acts as an acceptor site for -PO_2_O-H. The calculated intramolecular OH⋯O distances range from 1.50 to 1.67 Å, and are in agreement with literature [27].

As already seen from the chemical shift data, assignment of p*K*_a_ values to the two carboxyl groups 1′ and 2′ is difficult. The DFT-calculated energy for H_3_L^2−^ is lowest for H^+^ abstraction from 1′ COOH, while deprotonation of 2′ COOH is lowest in energy not before HL^4^^−^, i.e., for deprotonation of H_2_L^3−^ (see Table 2), in line with its calculated long-persisting hydrogen bond. The increased electron density due to close proximity of 1′ carboxylate as well as the phosphonate group with strong hydrogen bond acceptor properties impedes H^+^ abstraction from carboxylic group 2′.

Although DFT calculations suggest deprotonation of the trianionic species at the remaining functional groups very close in energy, from ^13^C NMR data the p*K*_a3_ can clearly be assigned to 4′, since the data of both the carboxyl carbon and the carbon of the adjacent methylene group (C4) reveal only one clear sigmoidal. This site can be considered independent from the other functional groups.

As implied by DFT, 4′ is not involved in intramolecular hydrogen bonding. ^1^H nuclear Overhauser enhancement spectroscopy (NOESY) proves an ‘elongated’ aliphatic carbon chain C2–C3–C4–COO(H). Distance- (*r*^–6^) and orientation-dependent NOE contacts (ellipses and arrows in Figure 6B,C), observable for H1b to both H4(a/b) but not to H3(a/b), indicate that the latter points in the opposite direction of H1b and are rather distant, whereas H1b points towards H4(a/b) and is closer in space. Assignment of 1a and 1b is straightforward by means of ^3^*J*_H,P_, where the larger coupling indicates the *transoid* arrangement between P and H1b whereas the smaller coupling is indicative of the *cisoid* arrangement for P and H1a (see horizontal projection in Figure 6B). Between pH 2 and 8, the signals due to H1a and H1b reflect the electronic situation in their respective half spaces well. H1a senses deprotonation (and changing hydrogen bonding situation) for the phosphonate as well as 1′ and 2′, and thus is successively shielded. As H1b points in the opposite direction of H1a and is too distant to sense alterations at 4′, its chemical environment is almost unaffected in that pH range.

^2^*J*_C2′,P_‘s minimum observed at pH = 5 (and possibly that of ^2^*J*_1a,1b_, too) is interpreted as the onset of H_2_L^3−^ deprotonation (emerging curve of HL^4−^, Figure 3); that is, abstraction of the 2′ carboxyl proton along with cleavage of the associated intramolecular hydrogen bond, almost exactly one pH unit below the corresponding p*K*_a_ value (6.13).

At pH/pD~8, upon emergence of the fully deprotonated species, the additional negative charge at the phosphonate group once again causes conformation changes because of intramolecular electrostatic repulsion among the phosphonate group and 1′ and 2′ carboxylate groups as well as dissociation of the remaining hydrogen bond. This process is clearly reflected by the simultaneous but opposite Karplus-like pH-dependent ^3^*J*_C,P_ curves, indicative of extensive charge-induced alterations in P–C2–C1–C1′ and P–C2–C3–C4 dihedral angles.

The angular dependence of P–O–C–C coupling has clearly been demonstrated for cyclic nucleotides, cyclic phosphites, and phosphates, and an almost symmetrical Karplus curve was found for P–C–C–C coupling in phosphine oxides. For acyclic organic phosphonates, ^1^*J*_C,P_ values have been determined as well [28,29,30,31,32,33,34,35,36]. Already in 1976, Buchanan and Benezra addressed the question whether phosphonates show a Karplus-type relationship in P–C–C–C coupling [37]. They draw the conclusion that relating ^3^*J*_C,P_ couplings to dihedral angles must be exercised with great care as they are very sensitive to electronic and steric substituent effects, and differ notably from geometrical requirements observed in corresponding *vicinal* ^3^*J*_H,H_ coupling.

The presented data on homo- and heteronuclear coupling constants might be a good basis for benchmarking DFT-calculated *J* values as demonstrated elsewhere [38]. Although the present study cannot cover quantitative information on the Karplus relationship of ^3^*J*_C,P_ in PBTC, it still adds to the examples of qualitatively demonstrating the value of coupling constants in understanding charge-induced alterations in the molecule’s conformation.

## 4. Materials and Methods

### 4.1. Starting Material

Aqueous PBTC solution can be obtained from various suppliers (from dedicated chemical distributors to specialty suppliers for the industrial applications). Most of these suppliers guarantee a PBTC content of 48–52%, but do not provide any further information regarding the purity of the solution (except SigmaAldrich, St. Louis, MO, USA; here a purity of >95% was specified but could not be confirmed; refer to Supplementary Materials). Already Armbruster et al. [10] reported on the several impurities of technical PBTC, depending on the conditions during synthesis and workup. Contaminants comprise, among others, phosphorous acid, ortho-phosphoric acid, phosphonopropionic, and phosphonosuccinic acids. Therefore, PBTC solutions from three different suppliers have been tested for their purity. The batch of highest purity, viz. 93% based on ^31^P signal integration, was used without further purification (TCI Germany GmbH, Eschborn, Germany; batch 1, see Supplementary Materials, Figure S5). Contained impurities do not affect p*K*_a_ determination since PBTC is the main component and NMR spectroscopy allows for precise examination of the signals associated with the molecule of interest. Assignment of the impurities was waived as their presence had no effect on the results of this study.

### 4.2. Sample Preparation

Three sample series (S1–S3) were prepared for NMR titration. S1 comprises samples prepared using deuterated chemicals and pD adjusted as described elsewhere [39]. Therefore, original 50% (*w*/*w*) aqueous PBTC solution, batch 1, TCI Germany GmbH (see Supplementary Materials) was adjusted to pH~6 using NaOH prior to lyophilization. The obtained pale brownish powder was then dissolved in D_2_O, yielding a 0.7 M stock solution with its concentration determined by signal integration of quantitative ^31^P NMR measurements of an aliquot spiked with a weighed amount of Na_2_HPO_4_. From this stock solution in D_2_O, sample solutions finally 350 mM in PBTC were prepared in the pD range 0–13.

Owing to the high sensitivity of ^31^P, but the comparably low sensitivity of ^13^C, corresponding series S2 and S3 were 1 mM and 25 mM in PBTC, respectively, covering pH ranges of 0–11 (S1) and 2–8 (S2) with increments of 0.2 to 0.4 pH units. Therefore, S2 and S3 were prepared by appropriate dilution of aliquots of the original 50% PBTC solution mentioned above using pure Milli-Q H_2_O (18.2 MΩ cm, Millipore, Burlington, MA, USA) for S2, and 10% D_2_O in Milli-Q H_2_O (*v*/*v*) for S3, as well as NaOH and HCl for pH adjustment with a pH meter (inoLab pH 730) equipped with a pH electrode (SCHOTT, BlueLine), respectively. The measured pH was checked using an ionic strength correction function according to the procedure described in [40]. In the case of *I*_m_ ≤ 0.5 m, the measured pH equals pH_c_ within uncertainties of 0.05 units. All chemicals for H_2_O solutions were p.a. grade and purchased from Roth (Arlesheim, Switzerland). In the case of S3, pH was corrected for deuterium. According to the common pD = pH(read) + 0.4 in pure D_2_O, and since the reading of the pH meter is very nearly a linear function of the atom-% deuterium [41], the used 10% D_2_O contents in S3 afford for addition of 0.04 pH units.

For the structure elucidation experiments, aliquots of the PBTC stock solution in D_2_O were diluted appropriately to yield 50 mM solutions, adjusted to pD = 1.9, 3.9, and 9.9, respectively.

### 4.3. NMR Spectroscopy

All NMR spectra were recorded at 25 °C with an Agilent DD2-600 NMR system, operating at 14.1 T with corresponding ^1^H, ^13^C, and ^31^P resonance frequencies of 599.8, 150.8, and 242.8 MHz, respectively, using a 5 mm oneNMR™ probe.

A total of 700 µL of D_2_O only (S1) or 90/10 (*v*/*v*) H_2_O/D_2_O solutions (S3), or 500 µL H_2_O solutions (S2) were transferred to 5 mm NMR tubes. In the case of series S2, the tubes contained a coaxial capillary insert filled with D_2_O for deuterium lock along with sodium 3-trimethylsilyl-propionate (TMSP-*d*_4_) and one droplet of 85% H_3_PO_4_ (Roth, *p.a.*) for external referencing.

^1^H NMR spectra (S1 and S2) were measured by accumulating 32 scans, using 2 s of acquisition time and relaxation delay, respectively, applying a 2 s pre-saturation pulse on the water resonance for water signal suppression. Spectra are referenced relative to the methyl signal of TMSP-*d*_4_ in D_2_O with *δ*_H_ = 0.00 ppm. ^1^H{^31^P} spectra (S3) were acquired analogously, with simultaneous broadband ^31^P-decoupling. For ^13^C NMR measurements (S3), 1024 scans were accumulated upon applying 4 s relaxation delay after a 3.3 µs excitation (30°) pulse and 1 s acquisition time, with ^1^H broadband-decoupling. ^31^P NMR spectra (S1 and S2) were obtained after excitation by a 30° pulse (4.16 µs), applying an acquisition time of 2 s, with simultaneous broadband ^1^H-decoupling. The relaxation delay between two excitations was 3 s. In case of quantitative ^31^P NMR spectra, 100 scans were accumulated upon excitation by a 30° pulse, inverse-gated ^1^H decoupling during the 3 s acquisition time, and relaxation delay of 60 s.

For unambiguous signal assignment, heteronuclear single-quantum coherence (HSQC) and heteronuclear multiple-bond correlation was performed using sequences with gradient-selection and adiabatic pulses. H,C-HSQC, H,C-HMBC, and H,P-HMBC spectra were acquired with 2 k × 512, 2 k × 1 k, and 2 k × 128 complex points in *F*_2_ and *F*_1_, with 24, 64, and 8 transitions per *F*_1_ increment, and a relaxation delay of 1 s, respectively. For polarization transfer, (2*J*)^−1^ delays of 4.0 and 41.7 ms were opted, corresponding to 125 Hz ^1^*J* in HSQC and 12 Hz ^n^*J* in either HMBC, respectively.

The NOESY spectrum was obtained from the S3 series’ pH 4.4 sample, acquired with 1 s pre-saturation, 500 ms mixing time, 2k × 512 complex points in *F*_2_ and *F*_1_, respectively, 64 transitions per *F*_1_ increment, and a relaxation delay of 1 s.

### 4.4. DFT Calculation

Using Gaussian16 program [42], density functional theory (DFT) calculations on PBTC species in different protonation states were performed at the B3LYP level in water through the use of polarizable continuum model (PCM) [25,39,43,44,45]. All-electron basis set of triple-zeta quality was used on all elements. Initial structures of the species were set to maximize the number of intramolecular hydrogen bonds since such structure tends to give energetically more stable complexes. Structures were confirmed to be at their energy minima through vibrational frequency analysis where no imaginary frequency was found to be present.

### 4.5. Data Processing Software

NMR spectra were processed with MestReNova, version 6.0.2., Mestrelab Research S.L. [46]. Creation of graphs for numerical data visualization and data fitting by non-linear sigmoidal dose–response fit algorithm was performed with Origin 2019, version 9.6.0.172, OriginLab Corporation. DFT-calculated structures are visualized with Avogadro [47].

## 5. Conclusions and Outlook

A comprehensive evaluation of multinuclear NMR chemical shift and coupling constant data, complemented by DFT calculations, allowed for a certain determination of PBTC’s p*K*_a_ values and the unambiguous assignment of the respective abstracted proton to the corresponding site among the phosphonic and carboxylic groups. These results endure both a reliable speciation and a good perception of the pH-dependent alterations in the molecule’s structure.

The used approach of NMR-based titration is superior to other methods, e.g. potentiometric titration, as its atomic resolution allows for the assignment of the origin of the abstracted proton. Unfortunately, especially for ^13^C NMR, the required concentrations in the 10–30 mM range are a clear disadvantage. With the higher sensitivity of ^1^H and ^31^P, especially in D_2_O solution, analyte concentrations of 100 µM or even lower are feasible.

The presented NMR spectra and p*K*_a_ values are critical spectroscopic and thermodynamic reference data, essential for determining molecular structures and stability constants of PBTC complexes and metal ions such as radionuclides.

The NMR data are also useful when dealing with PBTC modifications of any kind; for instance, PBTC derivatives such as its esters or, in long-term studies, its degradation products upon light-irradiation, thermal treatment, or other extreme conditions. Studies on PBTC in other environments are also conceivable, be it in organic solvents or in aqueous solutions with background electrolytes of different types or concentrations, or in solutions of other complex compositions.

## Figures and Tables

**Table 2:** Relative energies (in kcal mol^−1^) calculated from DFT-optimized structures (Figure 4) for a given macrospecies deprotonated at the respective functional groups.

Species		Carboxylic Groups		
	P* ^1^	1′	2′	4′
H_4_L^−^	±0.0	+26.8	+9.0	+25.3
H_3_L^2−^	-	±0.0	+7.6	+14.8
H_2_L^3−^	-	-	+7.7	±0.0
HL^4−^	+8.6	-	±0.0	-

^1^ P* denotes the phosphonate group.

## Data Availability

All data are included in this paper.

## Supplementary Materials

The following supporting information can be downloaded at: https://www.mdpi.com/article/10.3390/molecules27134067/s1, Figure S1: ^1^H,^31^P-HMBC, ^1^H,^13^C-HMBC, and ^1^H,^13^C-HSQC spectra of 50 mM PBTC D_2_O solution along with ^13^C signals showing the magnitudes of ^n^*J*_C,P;_ Figure S2: ^1^H, ^31^P{^1^H}, and ^13^C{^1^H} spectra of NMR pD-titration series of 350 mM PBTC in D_2_O solutions; Figure S3: Graphs of *δ*_P_ vs. pH, along with respective p*K*_a_ values obtained from sigmoidal dose–response fits, together with corresponding fitting parameters; Figure S4: Graph of ^3^*J*_C1′,P_ vs. pH along with corresponding sigmoidal bi-dose–response fit and corresponding fitting parameters; Table S1: Fitting parameters associated with sigmoidal (bi-)dose–response fits of the *δ*_P_ vs. pH graphs presented in Figure 3; Figures S5–S9: Quantitative ^1^H and ^31^P NMR spectra of PBTC solutions purchased from different suppliers, along with purity determined from ^31^P signal integration.

## Sample Availability

Samples of the compounds are not available from the authors.
