# Supplementary material for: 2-Phosphonobutane-1,2,4,-Tricarboxylic Acid (PBTC): pH-Dependent Behavior Studied by Means of Multinuclear NMR Spectroscopy
Source: Molecules. 2022 Jun 24;27(13):4067. doi: 10.3390/molecules27134067 (PMC9268528; doi:10.3390/molecules27134067)
Supplement: Supplementary file 1 [file molecules-27-04067-s001.zip › molecules-1775881-supplementary.pdf]

Supplementary Materials

2-Phosphonobutane-1,2,4,-tricarboxylic acid (PBTC): pH-dependent behavior studied by means of multinuclear NMR spectroscopy

Jerome Kretzschmar [1,*], Anne Wollenberg [2], Satoru Tsushima [1,3], Katja Schmeide [1] and Margret Acker [4]

[1] Helmholtz-Zentrum Dresden-Rossendorf, Institute of Resource Ecology, 01328 Dresden, Germany.

[2] Technical University Dresden, Professorship for Radiochemistry, Central Radionuclide Laboratory, 01062 Dresden, Germany.

[3] International Research Frontiers Initiative, Institute of Innovative Research, Tokyo Institute of Technology, Meguro, Tokyo, Japan.

[4] Technical University Dresden, Radiation Protection, Central Radionuclide Laboratory, 01062 Dresden, Germany.

* Correspondence: j.kretzschmar@hzdr.de

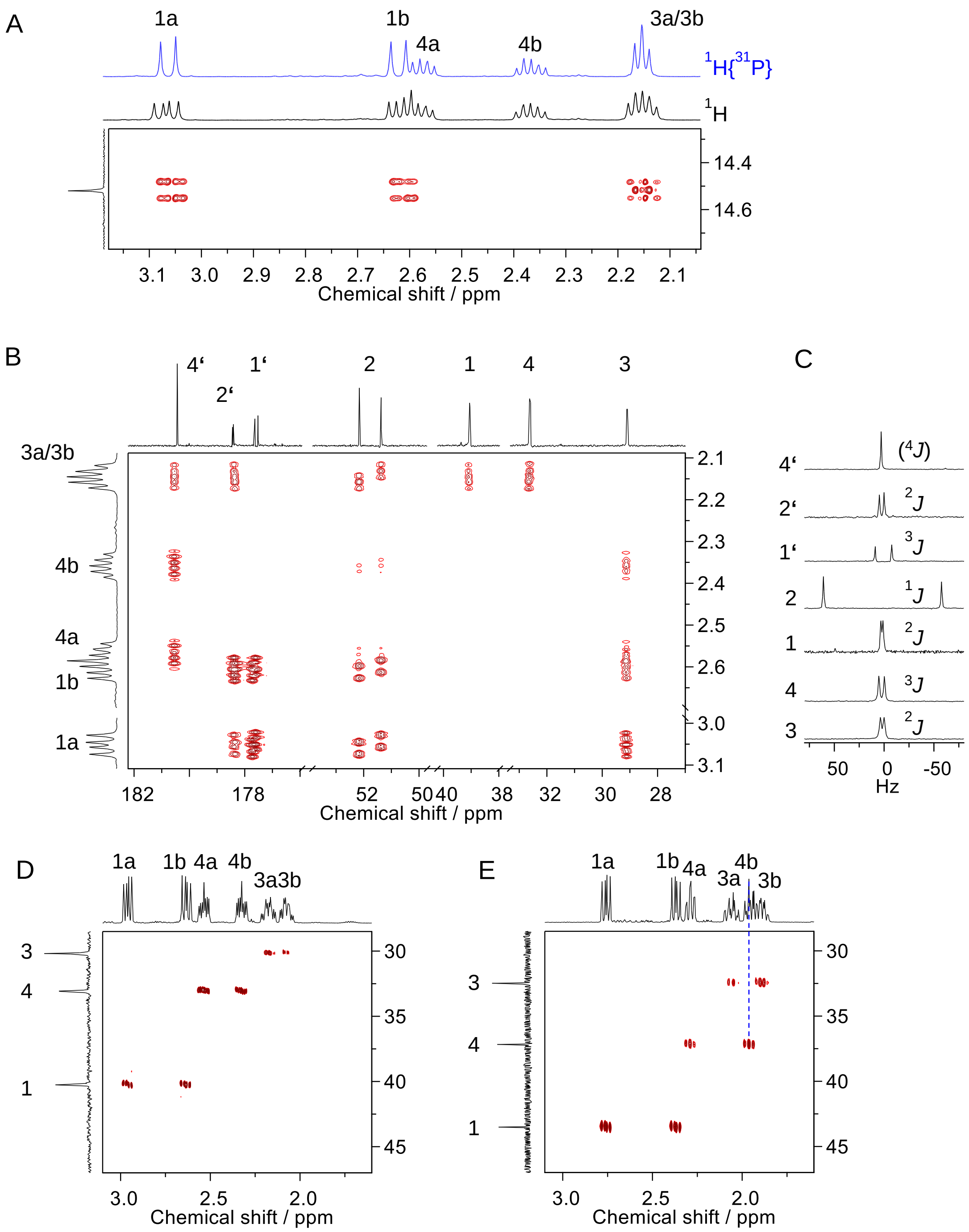

Figure S1. (A) ^{1}H,^{31}P-HMBC spectrum of 50 mM PBTC D_2O solution at pD = 1.9 along with the one-dimensional ^{1}H and ^{31}P broadband-decoupled ^{1}H NMR spectra as well as the ^{1}H broadband-decoupled ^{31}P NMR spectra as horizontal and vertical projections, respectively. (B) Transposed ^{1}H,^{13}C-HMBC spectrum of 50 mM PBTC D_2O solution at pD = 1.9 along with the one-dimensional ^{1}H broadband-decoupled ^{13}C NMR and ^{1}H NMR spectra as horizontal and vertical projections, respectively. (C) Expansion of the ^{13}C signals showing the magnitudes of $^{n}J_{C,P}$. ^{1}H,^{13}C-HSQC spectra of 50 mM PBTC D_2O solution at pD = 3.9 (D) and pD = 9.9 (E) along with the one-dimensional ^{1}H broadband-decoupled ^{13}C NMR and ^{1}H NMR spectra as horizontal and vertical projections, respectively. Labeling is according to Figure 1A.

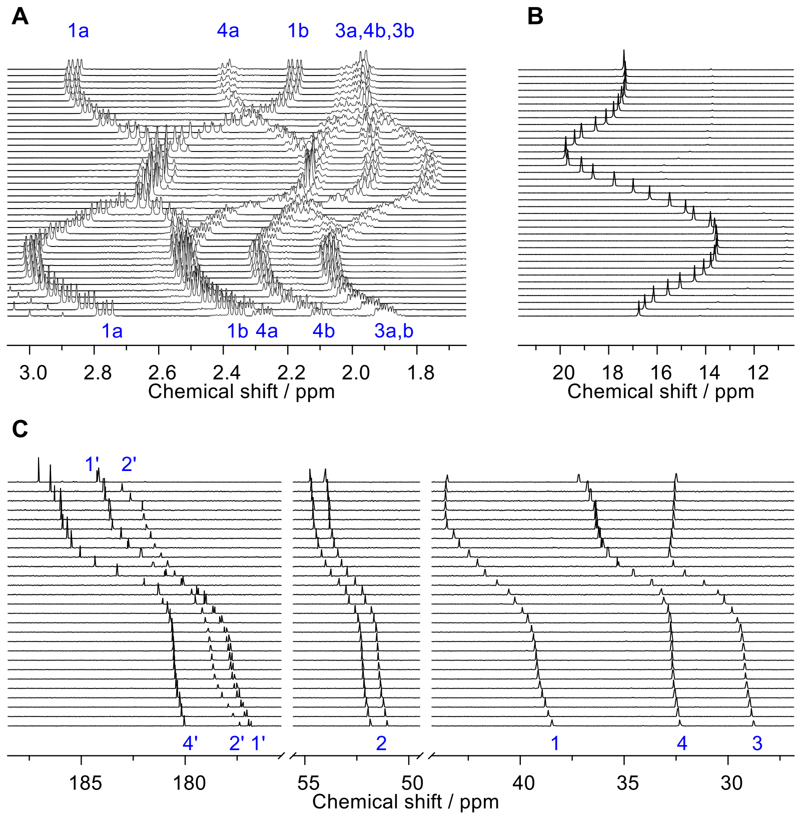

Figure S2. NMR pD-titration series of 350 mM PBTC in D_2O solutions. ^{1}H (A) and $^{31}P\{^{1}H\}$ (B) NMR spectra obtained in the pD range 0–13. (C) $^{13}C\{^{1}H\}$ NMR spectra in the pD range 1–10; pD values increase from bottom to top with increments of 0.2 to 0.4 units. For clarity, only spectral regions of interest are shown.

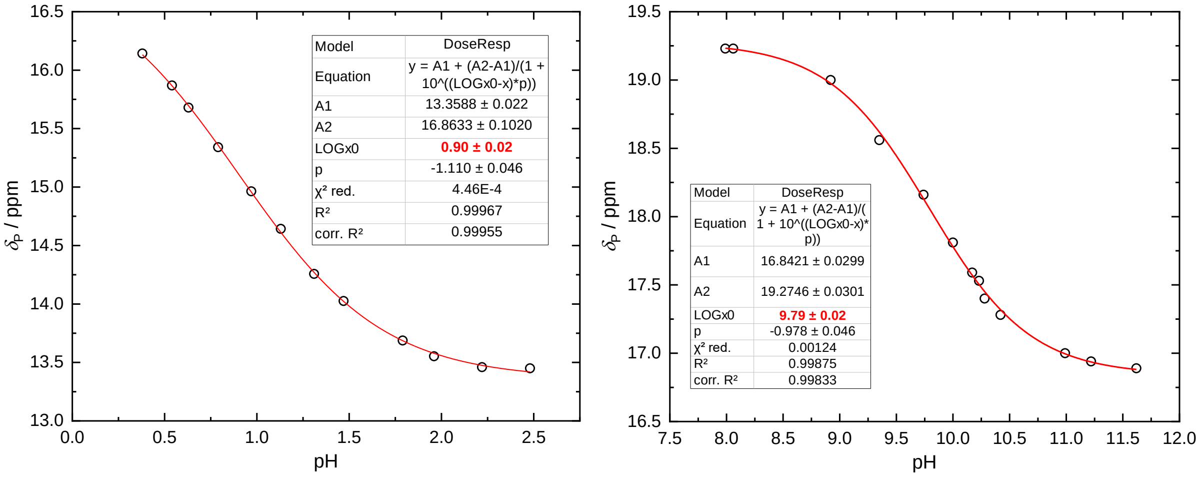

Figure S3. Graphs of pH-dependent ^{31}P NMR chemical shift values (black circles) based on the spectra shown in Figure 1D, along with respective pK_a values obtained from sigmoidal dose–response fits (red lines) together with corresponding fitting parameters.

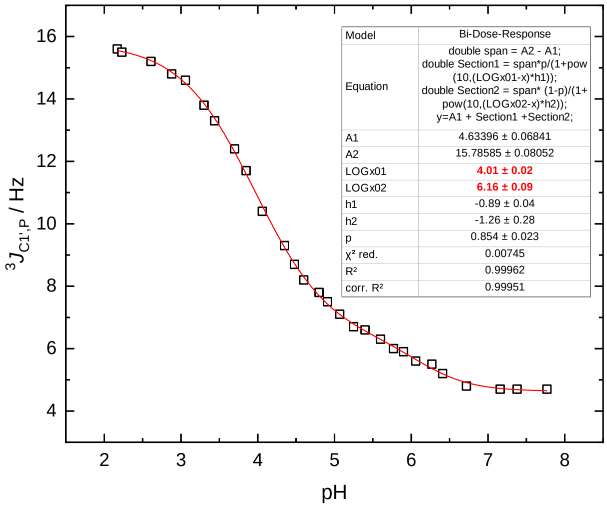

Figure S4. Graph of pH-dependent $^3J_{C1',P}$ values (black squares) obtained from $^{13}C\{^1H\}$ NMR spectra shown in Figure 5A, and corresponding sigmoidal bi-dose–response fit (red line) along with fitting parameters.

Table S1. Fitting parameters associated with sigmoidal (bi-)dose–response fits of the pH-dependent ^{13}C NMR chemical shift values presented in Figure 2.

Plot	**C1'**	**C2'**	**C1**	**C2**	**C4'**	**C4**
Model	Bi-Dose–Response				Dose–Response	
Equation	double span = A2 – A1;<br>double Section1 = span*p/(1+pow(10,(LOGx01–x)*h1));<br>double Section2 = span*(1–p)/(1+pow(10(LOGx02–x)*h2));<br>y=A1 + Section1 + Section2				y = A1 + (A2-A1)/(1 + 10^((LOGx0-x)*p))	
A1	178.229 ± 0.021	178.909 ± 0.010	39.531 ± 0.013	52.439 ± 0.008	180.629 ± 0.040	32.827 ± 0.024
A2	183.755 ± 0.025	181.925 ± 0.013	43.627 ± 0.015	54.711 ± 0.010	185.830 ± 0.037	36.326 ± 0.022
LOGx01	**3.92 ± 0.02**	**3.92 ± 0.02**	**3.82 ± 0.012**	**4.05 ± 0.02**	**4.76 ± 0.02**	**4.75 ± 0.02**
LOGx02	**6.13 ± 0.03**	**6.14 ± 0.03**	**6.14 ± 0.02**	**6.08 ± 0.05**	—	—
h1	0.95 ± 0.03	0.91 ± 0.03	1.02 ± 0.03	0.900 ± 0.026	—	—
h2	0.95 ± 0.05	0.88 ± 0.04	1.01 ± 0.04	0.89 ± 0.07	—	—
p	0.605 ± 0.016	0.598 ± 0.016	0.585 ± 0.010	0.68 ± 0.02	0.76 ± 0.02	0.80 ± 0.02
χ^2 red.	5.01E-04	9.11E-05	2.00E-04	6.39E-05	4.87E-03	1.94E-03
R^2	0.99988	0.99993	0.99991	0.99992	0.99889	0.99906
corr. R^2	0.99985	0.9999	0.99988	0.99989	0.99875	0.99894

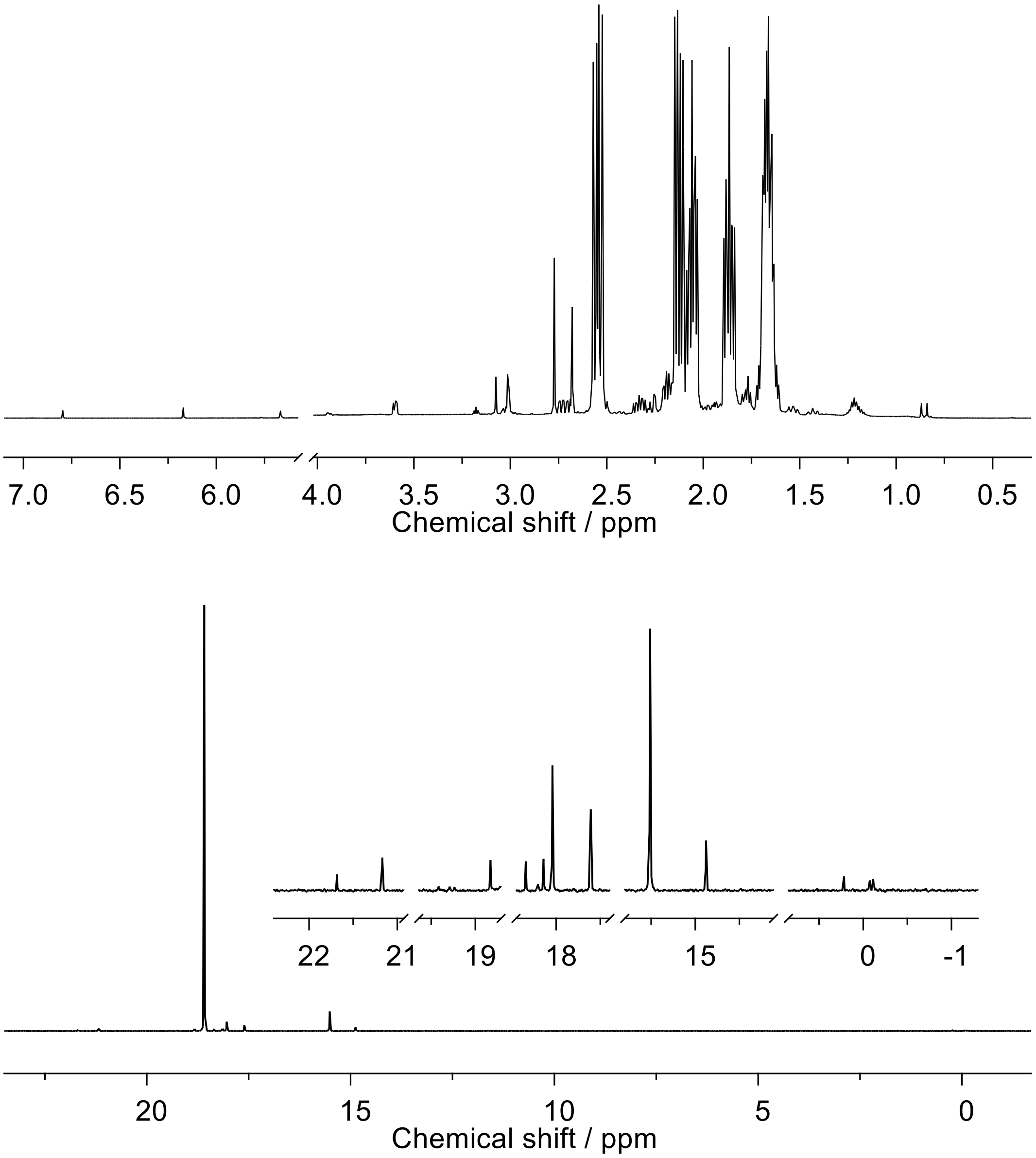

Figure S5. Quantitative ^{1}H (top) and ^{31}P NMR spectra (bottom, along with expansion as insert) of PBTC obtained from TCI Deutschland GmbH; batch 1 (used for the present study), LOT-no. GSDIJTH. Purity determined from ^{31}P signal integration was 93%.

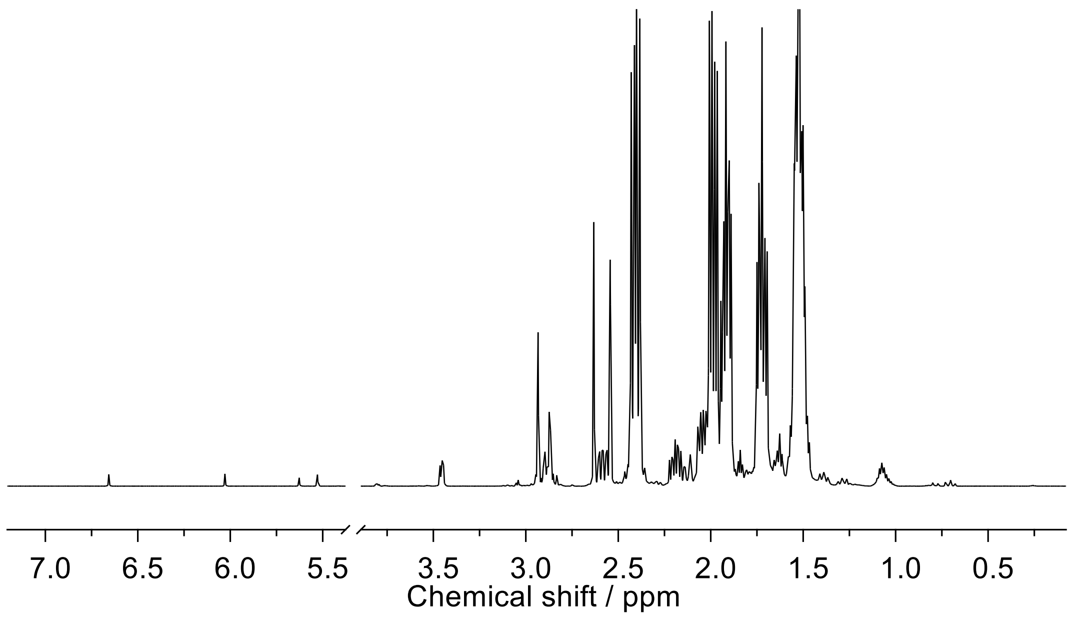

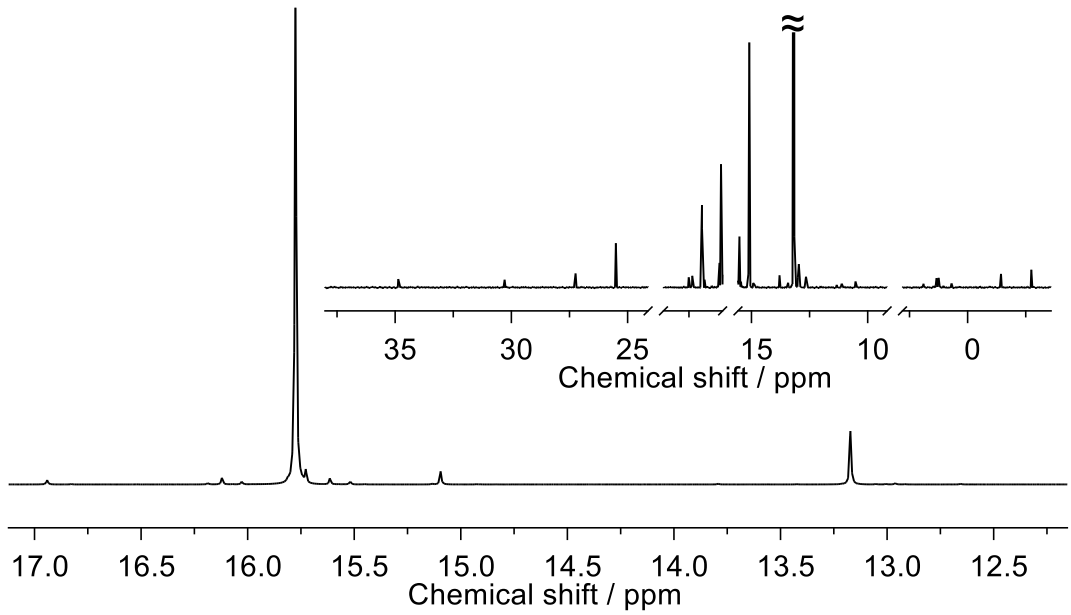

Figure S6. Quantitative ^{1}H (top) and ^{31}P NMR spectra (bottom, along with expansion as insert) of PBTC obtained from TCI Deutschland GmbH, batch 2, LOT-no. V8R4GEG. Purity determined from ^{31}P signal integration was 86%.

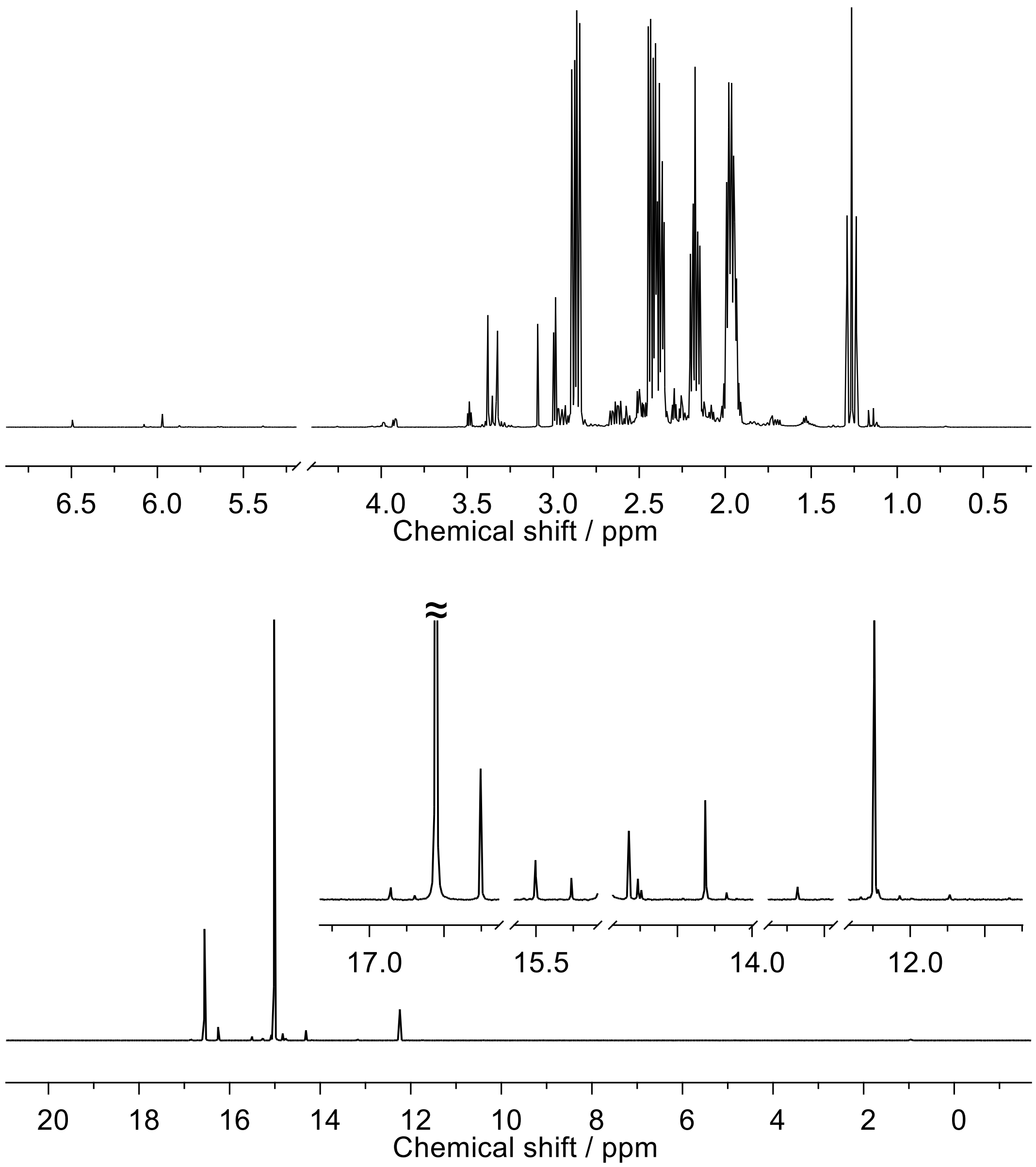

Figure S7. Quantitative ^{1}H (top) and ^{31}P NMR spectra (bottom, along with expansion as insert) of PBTC obtained from SigmaAldrich, LOT-no. 013V; MatrixScientific, Columbia, USA). Purity determined from ^{31}P signal integration was 67%.

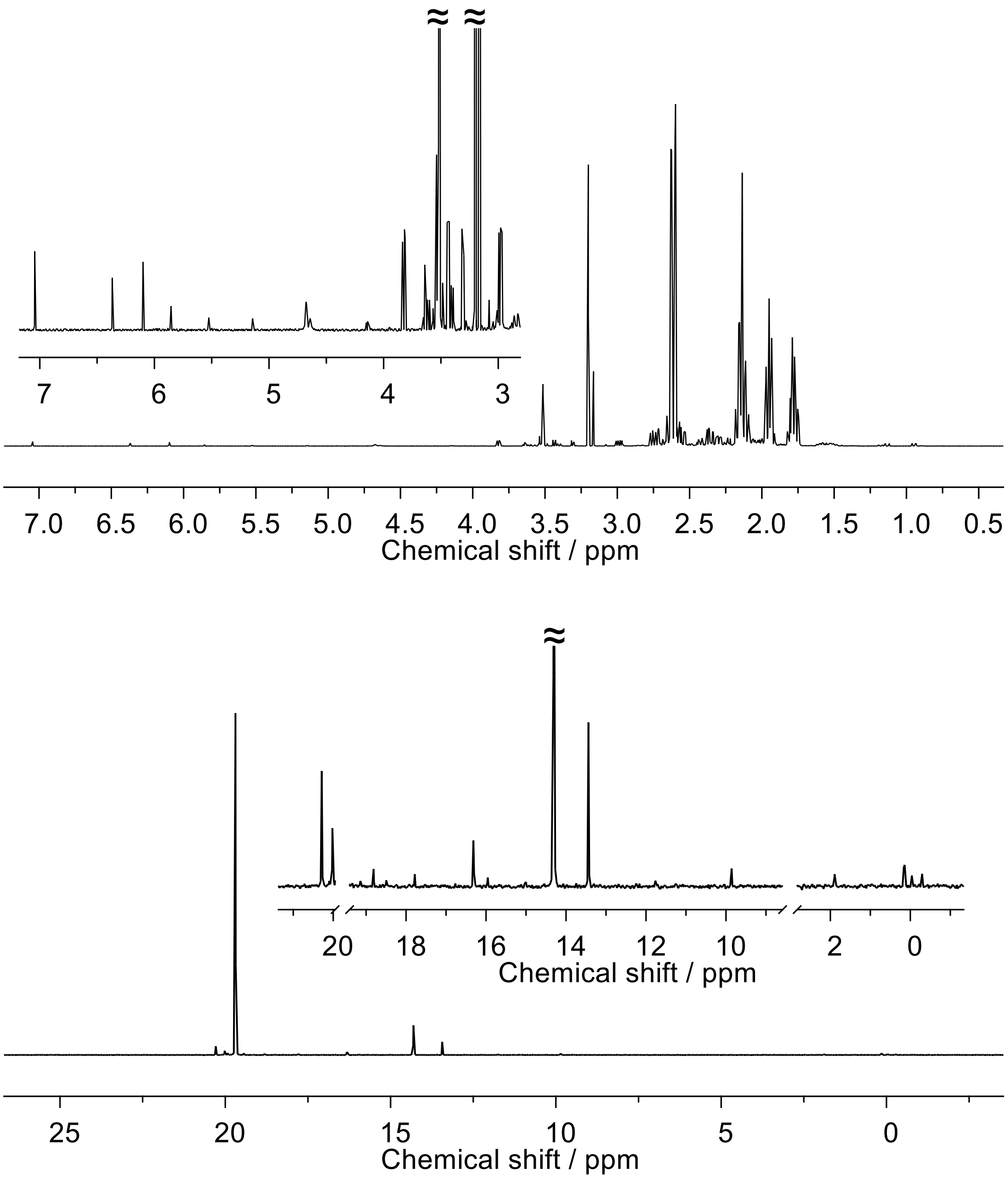

Figure S8. Quantitative ^{1}H (top, along with expansion as insert) and ^{31}P NMR spectra (bottom, along with expansion as insert) of PBTC-Na salt solution (CUBLEN P4014) obtained from Zschimmer & Schwarz Chemie GmbH, LOT-no. 78193. Purity determined from ^{31}P signal integration was 80%.

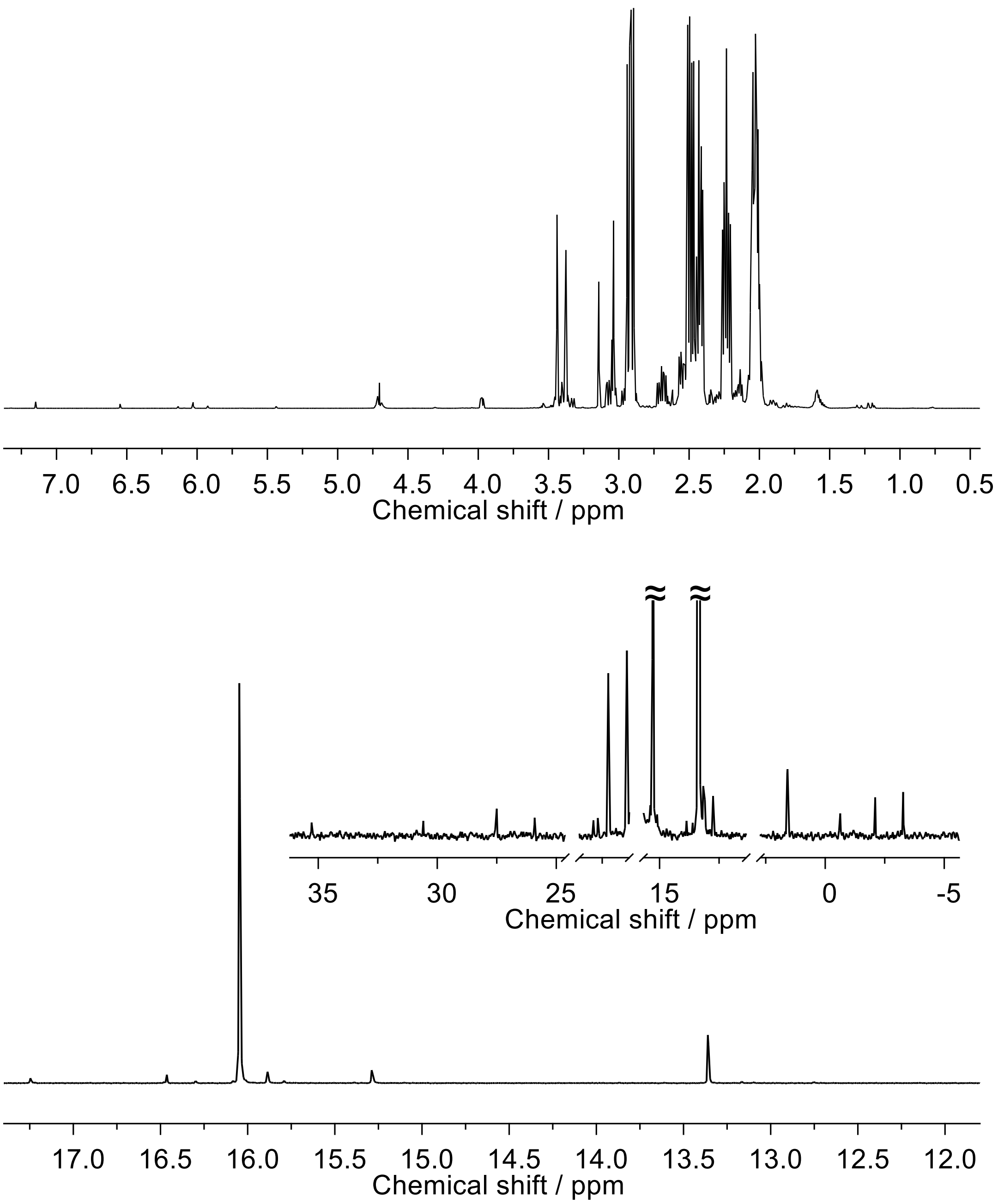

Figure S9. Quantitative ^{1}H (top) and ^{31}P NMR spectra (bottom, along with expansion as insert) of PBTC solution (CUBLEN P50) obtained from Zschimmer & Schwarz Chemie GmbH, LOT-no. 883806. Purity determined from ^{31}P signal integration was 82%.
